# Low frequency of moaA3 gene among the clinical isolates of *Mycobacterium tuberculosis *from Tamil Nadu and Pondicherry – south eastern coastal states of India

**DOI:** 10.1186/1471-2334-9-114

**Published:** 2009-07-25

**Authors:** Balaraman Sekar, Kamalanathan Arunagiri, Nagamiah Selvakumar, Kaluvuri Serena Preethi, Kandhaswami Menaka

**Affiliations:** 1 Division of Laboratories, Central Leprosy Teaching and Research Institute, Chengalpattu, Tamil Nadu, India; 2 Department of Mycobacteriology, Tuberculosis Research Centre, Chennai, Tamil Nadu, India

## Abstract

**Background:**

Comparative genomic analysis of *M. tuberculosis *H37Rv and *M. bovis *BCG have shown that 16 RDs (Regions of Differences) are deleted in BCG and have shown six deletion regions in *M. tuberculosis *H37Rv. RD1, is present in *M. tuberculosis *but is absent in all *M. bovis *BCG sub-strains. A study from Kerala, a south-western coastal state of India aimed to find out differences in RD1 region showed for the first time the presence of moaA3 gene in majority of their clinical isolates, that was absent in type strain H37Rv. We attempted to find out such polymorphism between type strains and the clinical isolates within RD1, targeting moaA3 gene among the clinical isolates of Tamil Nadu & Pondicherry, south-eastern coastal states of India

**Methods:**

One hundred and sixteen clinical isolates of *M. tuberculosis *were included in the study. PCR using RD1DLa and RD1DRa primers was carried out to amplify a 652 bp fragment, encoding for *cfp*10 and *esat *6 proteins of RD1. A second PCR using primers designed from the surrounding regions of *moa*A3 gene was done to confirm the presence of the full Open Reading Frame (ORF) in clinical isolates.

**Results:**

In *M. tuberculosis *H37Rv the expected 652 bp band was present. In BCG it was absent as expected, but a 386 bp fragment was amplified. Around 12/116 (10.3%) of our clinical isolates showed both 652 and 386 bp fragments. The additional 386 bp amplicon is a part of the *moaA3 *gene which codes for molybdopterin cofactor protein A in *M. bovis*. The second PCR amplified the flanking sequence of *moaA3 *and yielded the expected amplicon of 1254 bp in all those 10.3% of clinical isolates which had the 386 bp fragment. However the earlier study carried out in Kerala, reported the presence of *moaA3 *gene in majority (97%) of their clinical isolates.

**Conclusion:**

This finding showed that there was regional variation presenting polymorphism in *moA3 *gene, among the strains of *M. tuberculosis *and further strengthens the speculation of genetic differences among the strains of Kerala and Tamil Nadu & Pondicherry, the South Indian states

## Background

The passaging of *M. tuberculosis *H37Rv and *M. bovis *BCG over years brought out changes in their genomic and virulence characteristics. Mahairas et al described the genomic changes in Region of difference 1 (RD1) – a region that is present in all virulent laboratory and clinical strains of *Mycobacterium bovis *and *Mycobacterium tuberculosis *[1]. The loss of RD1 in *M. bovis *BCG, contributes to the attenuation and its reintroduction into an attenuated strain resulted in a significant increase in virulence [2]. Deletions of RvD1 to RvD5 and TbD1 in H37 Rv are also reported [3].

A study was planned previously to screen the clinical isolates of *M. tuberculosis *from Kerala, a south-western costal state of South India for differences in RD1 region [4]. Amplification using primers that span the ORF coding for *cfp*10 and *esat *6 has shown to have produced 652 bp in all *M. tuberculosis *clinical isolates studied and also an extra amplicon of 386 bp was amplified in 97% of local field isolates, but not in *M*. *tuberculosis *H37 Rv. Further analysis revealed that the primer that was made spanning the RD1 region was similar to the portions of the *moaA3 *gene in the RvD5 region, which resulted in the amplification of 386 bp fragment. This amplicon spanned the nucleotides 575 to 960 of the *moaA3 *gene in *M*. *bovis*

This prompted us to screen the local isolates from states of Tamil Nadu and Pondicherry, on the south-eastern coastal region of South India for the presence of *moaA3 gene*. Our study revealed a low frequency of *moaA3 *gene among Tamil Nadu and Pondicherry clinical isolates compared to higher frequency of *moaA3 *among the clinical isolates from the neighbouring Kerala state.

## Methods

### Mycobacterial Strains

In this study, we included 116 *M*.*tuberculosis *non-repeat clinical isolates from different parts of Tamil Nadu and Pondicherry. (74 – Obtained from Tuberculosis Research Centre (TRC), Chennai, Tamil Nadu and 42 from Government Chest Disease Hospital, Pondicherry). All the strains included in this study were isolated in the year 2006 and the analysis was completed by April 2007. *M*. *tuberculosis *H37RV and *M*.*bovis *BCG were used as type strains. All the strains were identified by biochemical analysis.

Since this study was based on laboratory work carried out with mycobacterial strains and there was no experimental work involving human (or) animal, the ethical approval was not required.

### Drug susceptibility Testing (DST)

The in-vitro DST was carried out on solid Lowenstein-Jensen (LJ) medium using the Minimal Inhibitory Concentration (MIC) method/Resistance ratio (RR) method [5].

Briefly a 3 mm loopful of the bacterial suspension containing approximately 4 mg/ml was used to inoculate drug-free and drug-containing slopes (32, 64 and 128 mg/ml). The standard strain, *M. tuberculosis *H37 Rv was included in every batch of tests as a check on the inoculumn size as well as the drug concentrations in the medium. The slopes were incubated at 37°C and read at the end of 28 days. The lowest drug concentration which inhibited growth (defined as 20 colonies) was taken as the MIC. RR was defined as the ratio of the MIC of the test isolate to the MIC of the reference strain H37Rv.

### Extraction of DNA from clinical samples

All the samples were processed for DNA extraction as per the standard method of Herman et al [6]. Essentially, the cells were lysed with lysozyme followed by treatment with proteinase K and sodium dodecyl sulphate. Proteins and macromolecules were precipitated using NaCl and hexadecyltrimethylammonium bromide -NaCl solutions. Nucleic acids were recovered from aqueous phase after extraction with chloroform and isoamyl alcohol. DNA was further precipitated 30 minutes with isopropanol at -20°C. The pellet was washed with ethanol and later reconstituted in TE buffer.

### PCR amplification of RD1 region

PCR amplification of the RD1 was carried out as described earlier [4]. Briefly Primers RD1 DLa (For):-5'-AGA TGA AGA CCG ATG CCG CTA C-3', RD1 DRa (Rev):-5'-CCC GTG TTT CGC TAT TCT ACG C-3' that can span the RD1 region Rv3874 and Rv3875 coding for *cfp 10 *and *esat 6 *were used. After initial denaturation, amplification was performed in PCR thermocycler (Minicycler-MJ Research) for 35 cycles at 95°C/40 sec, 64°C/1 min, 72°C/1 min followed by a final extension at 72°C/5 min. It was expected to yield a 652 bp product and also 386 bp products if *moaA3 *was present in the test isolates, only 652 bp product in *M. tuberculosis *H37Rv and only 386 bp products in *M. bovis *BCG.

To identify the flanking sequence of the 386 bp another set of primers were used (moaFP – 5'-CCC ATC GTG GTC GTT CAC C-3' and moaRP – 5'-CGA TGG CAG CGG TTT ACA G-3') to amplify a 1254 bp product, with the same conditions as done for the above amplification.

### PCR amplification for IS6110

Amplification of DNA for IS6110 was performed with primers IS-F – 5'-CCTGCGAGCGTA GG CGTCGG-3'and IS-R – 5'CTCGTCCAGCGCCGCTTCGG-3', to amplify 123 bp fragment of insertion element IS6110 of *M. tuberculosis *complex as reported earlier [7]. Briefly PCR was carried out in 50 μl volume, using 200 μM dNTPs, 20 pM of each primer and 1 U of Taq polymerase, followed by template. Conditions followed were initial denaturation at 94°C for 4 minutes, followed by 35 cycles at 90°C for 1 minute, 60°C for 1 minute, and 72°C for 1 minute, and a final extension at 72°C for 10 minutes.

## Results

### Screening of RD1

**Figure 1:**
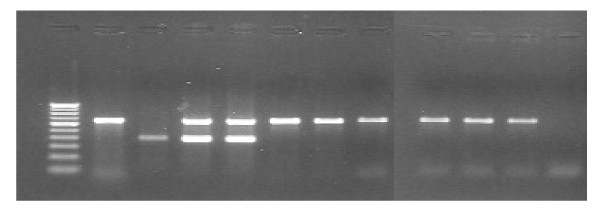
**PCR for amplification of *cfp *10 and *esat *6 of RD1**. Lane-1 – Molecular weight marker (100 bp ladder). Lane-2 – *M. tuberculosis *H37Rv, showing only 652 bp (RD1). Lane-3 – *M. bovis *BCG showing only 386 bp. Lane-4 & 5 – Clinical isolates showing 652 bp & 386 bp extra amplicon (moaA3). Lane-6 to 11 – Clinical isolates showing 652 bp only. Lane12 – Negative control.

The PCR primers designed to amplify regions within RD1 to find out the polymorphism between the type strain and the clinical isolates amplified the expected 652 bp fragment (comprising of Rv 3874 and Rv 3875, coding for *cfp*10 and *esat *6) in *M*. *tuberculosis *H37Rv. In *M*.*bovis *BCG, the 652 bp was absent as expected, but a 386 bp fragment was amplified. All the 116 clinical isolates showed 652 bp products. However in 8 out of 74 (10.8%) of Tamil Nadu isolates and 4 out of 42 (9.5%) of Pondicherry isolates showed both 652 and 386 bp fragments (Figure 1). Thus, 12 out of the 116(10.3%) samples showed the extra amplicon of 386 bp. (Table 1)

**Table 1:** PCR for amplification of *cfp10 *and *esat *6 of RD1

Source of samples	No. of samples investigated	No. of samples positive for IS6110	No. of samples positive for 652 bp	No. of samples positive for 652 bp + 386 bp
Tamil Nadu	74	74 (100%)	74 (100%)	08 (10.8%)
Pondicherry	42	42 (100%)	42 (100%)	04 (9.5%)
Total	116	116 (100%)	116 (100%)	12 (10.3%)

652 bp – product found in *M. tuberculosis *H37Rv; 386 bp – product found in M. bovis BCG

**Figure 2:**
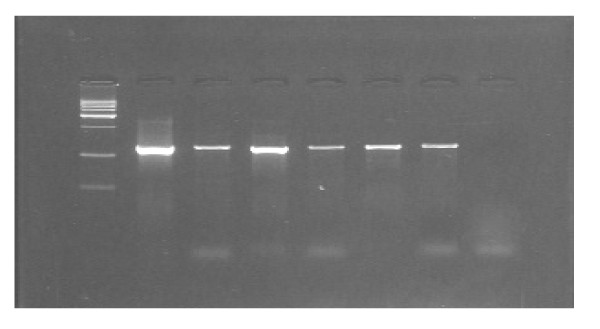
**PCR for amplification of flanking sequence of 386 bp of moaA3 gene**. Lane-1 – Molecular weight marker (500 bp ladder). Lane-2 to 7 – Clinical isolates which were positive for 386 bp extra amplicon, showing 1254 bp of flanking sequence. Lane-8 – Negative control.

The second PCR using primers designed from the surrounding region of *moaA3 *gene done to confirm the presence of the full ORF in clinical isolates, amplified the expected 1254 bp in all the clinical isolates which showed the extra 386 bp amplicon. (Figure 2) (Table 2)

**Table 2:** PCR for amplification of Flanking sequence of 386 bp of moaA3 gene

Source of samples	No. of samples investigated	No. of samples positive for 652 bp +386 bp	No. of samples positive for Flanking sequence among samples showing 386 bp amplicon
Tamil Nadu	74	08 (10.8%)	08 (100%)
Pondicherry	42	04 (9.5%)	04(100%)
Total	116	12 (10.3%)	12 (100%)

386 bp product found in *M. bovis *BCG & in some field isolates

1254 bp product coded flanking sequence of 386 bp fragment of moaA3 gene

As our isolates showed a lower frequency of *moaA3 *gene, we checked the possibility of false negativity by including 50 DNA templates from those isolates which were positive for 652 bp of RD1, but found negative for 386 bp extra amplicon and ran second PCR to amplify the flanking sequences. None of these templates were positive for flanking sequence. Thus, the remote possibility of false negativity as a cause for lower frequency of *moaA3 *gene among our isolates was ruled out.

### Screening of IS 6110

PCR for IS6110 showed the 123 bp fragment in all 116 (100%) clinical isolates. (Table 1)

### Correlation with drug susceptibility results

For 58 TRC clinical isolates, drug susceptibility result was available. Resistance to Streptomycin (S), Isoniazid (H), Rifampicin(R), and Ethambutol (E) were 33%, 14%, 0% and 25%, among *moaA3 *positive samples where as among *moaA3 *negative samples were 29%, 34%, 20% and 14% respectively. Multi Drug Resistance (MDR) was observed in 18% (9/46) of samples negative for *moaA3*, where as no MDR was observed among *moaA3 *positive sample. However, as most of the samples tested were *moaA3 *negative and only few samples were positive the statistical significance could not be drawn for want of adequate sample size in both the comparable groups.

## Discussion

Studies with subtractive hybridization and microarrays have identified 16 regions present in *M. tuberculosis *H37Rv but are absent in *M*. *bovis *BCG [1,8]. Further deletions of RvD1 to RvD5 and TbD1 genes in H37Rv are also reported [3]. RD1 region comprises of nine genes (Rv3871 to Rv3879c) and spans a 9.5-kb region. In *M. bovis *BCG, RD1 deletion completely removes seven genes (Rv3872 to Rv3878) and truncates two others (Rv3871 and Rv3879c) [9].

Of the nine genes predicted within the 9.5 kb RD1 region, those coding for *cfp *10 (Rv3874) and *esat *6 (Rv3875) are immunogenic and RD1 deletion mutants of *M. tuberculosis *have been found to be less virulent [9]

Thus, generation of deletion of genes appear to be a major mechanism for creating genetic diversity.

Based on this, a study from Kerala was carried-out to screen for differences in the RD1 region. Amplification using primers that span the ORF coding for *cfp*10 and *esat *6 was expected to give a 652 bp PCR product. But the PCR results revealed an extra amplicon of 386 bp in 97% of the clinical isolates screened. Further characterization by sequencing and homology search indicated that this region is a part of the *moaA3 *gene which encodes for molybdopterin cofactor protein A in *M*.*bovis*. The PCR primer that was made spanning the RD1 region was shown to be similar to portions of the *moaA3 *(MT3427) gene in the RvD5 region in clinical isolates and also in CDC1551 which resulted in the amplification of the 386 bp fragment. This amplicon spanned the nucleotides 575 to 960 of the *moaA3 *gene (MT 3355) in M. *bovis *[4]

With this background, we searched for the presence of *moaA3 *gene among the clinical isolates collected from Tamil Nadu and Pondicherry. Our search showed presence of 652 bp of *cfp*10 and *esat *6 of RD1 region in all the 116 clinical isolates screened but only a limited number of 12/116 (10.3%) clinical isolates showed the presence of 386 bp amplicon of *moaA3*. Amplification of flanking sequence of 386 bp *moaA3 *showed the expected 1254 bp product in among all the isolates showed the extra 386 bp amplicon. Thus, in contrast to Kerala study, only in limited proportion of our isolates *moaA3 *was amplified using the designed primer.

Although Rao et al, [10] reported total absence of RD1 region in clinical isolates, in a study from Hyderabad, Andhrapradesh (a south eastern coastal state) all our clinical isolates showed the presence of RD1 (*cfp *10 and *east *6) region. In fact, few isolates we obtained from Hyderabad were found positive for RD1 region in all the isolates (data not shown). This is in concurrence with other reports [11,4].

In H37Rv, the *moaA3 *amplicon was shown to be absent. The RvD5 region from which the amplicon was generated is an IS6110 mediated deletion in the type strain H37Rv [3]. IS6110 is a powerful genetic marker for strain differentiation [12]. In general low/no copies of IS6110 were implicated among south-east Asian strains, including India [13-15].

However, a closer scrutiny at the frequency of no/low copies of IS6110 reveals that they are commoner among Kerala strains than those from Tamil Nadu. An analysis from Kerala [16] reported about 24% of no copies and 39% of single copy of IS6110 in their isolates. Thus, around 62.5% of (50 out of 80) strains analyzed were not type-able by IS6110 based finger printing. This reported to be higher than the small numbers of IS6110 – deficient strains -1%–4% reported in Tamil Nadu [17,18]. It is appropriate to underscore that all our isolates screened for IS6110 by PCR were found positive.

Adding to this genetic variations, our finding of low frequency of 10.3% of *moaA3 *gene among Tamil Nadu and Pondicherry isolates compared to the higher proportion of 97% among Kerala isolates, further strengthens the speculation of genetic variation among the strains of Kerala and Tamil Nadu & Pondicherry, the South Indian states.

Further in screening of different genes of RD1 region among clinical isolates from Kerala and Western and Northern India, Rv 3871 and Rv 3872 (part of RD1 region) was reported as high as 98% in Kerala isolates, where as only 30% was reported in the other isolates screened [11].

This further emphasizes the need to carry out systematic molecular epidemiological studies in these endemic areas to explore any other genetic variations. Further the role of IS6110 or any other insertion sequences or mobile genetic elements in the genetic variation may also be investigated.

## Conclusion

Our attempt to screen for *moaA3 *gene among the clinical isolates of Tamil Nadu and Pondicherry revealed a low frequency of *moaA3 *gene compared to a high frequency of *moaA3 *gene among Kerala isolates. This finding showed a regional variation presenting polymorphism in *moA3 *gene, among the strains of *M*.*tuberculosis *and further strengthens the speculation of genetic variations among the strains of Kerala and Tamil Nadu & Pondicherry, the neighbouring South Indian states.

## List of abbreviations

RD: Region of Difference; BCG: Bacille Calmette-Guerin; *cfp*10: culture filtrate protein 10; *esat *6: early secreted antigenic target 6; ORF: Open Reading Frame; PCR: Polymerase Chain Reaction; MIC: Minimal Inhibitory Concentration; MDR: Multi Drug Resistance; DST: Drug susceptibility Testing.

## Competing interests

The authors declare that they have no competing interests.

## Authors' contributions

BS conceived, designed and supervised the study and carried out data analysis and wrote the manuscript. KA co-designed the study and carried out most of the experiment and assisted in the data analysis and preparation of the manuscript. NSK participated in the design and coordination of the study. KSP did part of the experimental work and coordinated. KM co-participated in most of the experiments and assisted in the data analysis

## Pre-publication history

The pre-publication history for this paper can be accessed here:

http://www.biomedcentral.com/1471-2334/9/114/prepub
